# The efficacy of rhythmic training for enhancing language and reading skills in children: a systematic review

**DOI:** 10.1186/s40359-026-04969-6

**Published:** 2026-07-11

**Authors:** Chiara Destro Pastizzaro, Lisa Toffoli, Giulia Stefanelli, Giovanni Mento, Vincenza Tarantino

**Affiliations:** 1 Department of Biomedicine, Neurosciences and Advanced Diagnostics, University of Palermo, Palermo, Italy; 2 NeuroDev Lab, Department of General Psychology, University of Padua, Padua, Italy; 3 Scientific Institute, IRCCS E. Medea, Conegliano, Treviso, Italy; 4 Department of Psychology, Educational Sciences and Human Movement, University of Palermo, Palermo, Italy

**Keywords:** Rhythm, Language disorder, Dyslexia, Speech, Reading, Literacy, Music, Rehabilitation, Synchronization, Entrainment

## Abstract

**Background:**

The human brain has the inherent ability to extract temporal regularities from sensory input and to synchronize with them. Language production and comprehension rely heavily on this fundamental ability. Rhythm-based training programs designed to boost language functions, especially across childhood, aim to facilitate the brain’s capacity to capture regular patterns in verbal streams. This systematic review critically evaluates the evidence on the efficacy of rhythmic training in improving linguistic skills in typically developing children as well as in children with language or reading difficulties.

**Methods:**

Following PRISMA guidelines, 21 studies published between 2009 and 2024 were selected, which assessed performance in linguistic tasks after training, in comparison with a control group or condition.

**Results:**

Twenty studies reported improved linguistic performance, mainly in phonological awareness and reading fluency (speed and accuracy). In particular, music-based rhythmic programs, which included activities such as producing movements synchronized to instruments, yielded effects comparable to validated phonological or reading training, with some studies also reporting gains in language-related cognitive and perceptual domains. Similarly, language-based rhythmic protocols, which provided verbal materials synchronized with instruments and primarily focused on children with dyslexia, reported improved reading skills. Furthermore, rhythmic priming studies demonstrated that even brief (17-second) exposures to regular rhythms enhanced subsequent syntactic processing compared to irregular rhythms. Interestingly, the effects emerged even in the absence of melodic or motor components.

**Conclusion:**

Overall, the findings of the reviewed studies highlight the potential of rhythmic training as a versatile tool for supporting language and reading development in children, including those with developmental language disorder or dyslexia. However, several methodological issues limit their interpretability. Before well-founded conclusions can be drawn, there is a substantial need to improve methodological rigor. Future studies should refine control conditions, standardize the range and types of outcome measures, and examine long-term effects.

**Supplementary Information:**

The online version contains supplementary material available at https://doi.org/10.1186/s40359-026-04969-6.

## Introduction

Numerous physiological processes in the human body, such as breathing, heartbeat, menstrual cycles, circadian rhythms, and neural activity, can be conceptualized as oscillatory systems characterized by intrinsic periodicity, which can synchronize with the periodicity of external phenomena [1]. The human brain is particularly optimized to capture external rhythmic patterns through two key neurocognitive mechanisms: statistical learning and neural entrainment. Statistical learning refers to the continuous tracking of probabilistic relationships between environmental inputs, even with mere exposure, which enables the brain to extract regularities in the occurrence of events and to learn associations between them [2–4]. Entrainment is an intrinsic property of the nervous system and refers to the natural temporal alignment of neural oscillations with external rhythms, which promotes synchronization and responses in phase with their temporal structure [5–7].

Language and music are among the most remarkable examples of how the brain excels in detecting statistical regularities and synchronizing with them, since both rely on a rhythmic organization [8]. Language inherently contains multiple cues that facilitate the extraction of repetitive patterns: sounds are organized into smaller chunks, such as phonemes, and larger units, including words and phrases, that occur with a certain regularity. Moreover, naturally produced speech is articulated in structured statistical patterns (e.g., transitional probabilities, non-adjacent dependencies, and co-occurrence of word-object pairs), which aid the brain in segmenting the continuous speech stream, thereby facilitating the detection of discrete linguistic units and enabling language comprehension and production [9]. Word stress and prosody within sentences are other cases of periodic linguistic patterns that the brain can readily recognize and anticipate [10]. Music offers the clearest example of temporal regularity, with rhythmic elements, such as tempo and meter, creating predictable structures. These rhythmic cues allow the brain to easily follow musical patterns [11] and readily synchronize motor actions to them, as seen especially in activities such as dance [12].

According to the Theory of Dynamic Attending [13], external stimuli with rhythmic regularities are decoded in the brain through entrainment of endogenous neural oscillations across multiple hierarchical levels [14]. These neural representations mediate the brain’s ability to extract external temporal regularities from events and, more remarkably, to predict, based on these associations, future events and to adjust to them [15]. All perceptual and cognitive processes may benefit from rhythmic entrainment [16, 17]. For example, auditory as well as visual stimuli are processed more efficiently and rapidly when presented on metrically salient beats, musical pulses, or expected time points [18–21].

Language learning particularly benefits from this brain ability, especially in early stages of development, when the brain exhibits heightened plasticity and pronounced sensitivity to external rhythmic and temporal regularities [22–24]. For instance, regular rhythms or rhythm primes have been shown to facilitate synchronization between external stimuli and internal neural oscillations, influencing subsequent speech perception and reading [25, 26], as well as syntactic and semantic processing in patients with language and temporal processing deficits [27, 28]. Furthermore, associations between early rhythmic skills and phonological and grammatical skills have been largely documented in preschool and school-aged children [29–31].

In line with this view, a unifying framework, the Processing Rhythm in Speech and Music [32], integrating elements from previous models [33–35], has demonstrated that speech/language and music share fundamental neurocognitive mechanisms, namely: (i) precise, fine-grained auditory processing; (ii) synchronization/entrainment of neural oscillations to external rhythmic stimuli; and (iii) sensorimotor coupling. Within this framework, rhythmic processing plays a driving role in both language and music rhythm processing and shows strong potential to support the development and enhancement of speech and language skills in children.

Importantly, atypical rhythm processing - encompassing deficits in beat perception, synchronization, or temporal prediction - constitutes a major risk factor for developmental language disorder (DLD) and developmental dyslexia (DD) [36]. DLD is characterized by deficits in language and/or speech comprehension and production [37]; DD, also referred to as specific reading disorder, is marked by inaccurate or non-fluent reading and poor word decoding [38]. Although DLD and DD are distinct disorders, they frequently co-occur [39]. This reflects the close and reciprocal link between early oral language skills and later literacy abilities [40], with phonological awareness and rapid automatized naming serving as predictors of subsequent word-level decoding and spelling [41–43]. Interestingly, according to the Atypical Rhythm Risk Hypothesis [36], one of the processes shared by both disorders is indeed rhythm processing; therefore, improvements in both spoken language and reading are expected when targeting rhythmic training in these populations.

Several studies have investigated whether musical and rhythmic training can enhance speech and aid in the rehabilitation of speech and language disorders, particularly in early childhood. In 2015, Gordon and colleagues [44] conducted a meta-analysis on the effects of music training on literacy skills, focusing on phonological awareness and reading fluency. They found significant benefits of music training for phonological awareness, with greater effects observed in children who received more hours of training. No consistent effects were found on reading fluency.

Based on the large evidence showing that rhythm facilitates statistical learning and brain entrainment mechanisms, the goal of the present study was to provide an updated systematic review of rhythm-based training research targeting language skill enhancement in children. We extended the work of Gordon et al. [44] by including both music-based and non-music-based rhythmic interventions. Furthermore, we considered multi-session training protocols, conducted in real-world settings (i.e., schools and clinics), as well as single-session training protocols, mostly conducted in laboratory settings. The following research questions were used to guide the screening of studies: (1) What types of rhythmic training have been used in the literature to boost linguistic functions? (2) What type of control groups or conditions have been employed? (3) Which linguistic functions have been tested as outcome measures? By addressing these questions, this review seeks to offer a critical evaluation of the efficacy of rhythm-based training in promoting language and reading skills.

## Methods

The Preferred Reporting Items for Systematic Reviews and Meta-Analyses (PRISMA) Statement [45] was applied, as described below.

### Eligibility criteria and search strategy

The eligible studies were peer-reviewed manuscripts published between 2009 and 2024 in English. We restricted the time frame to publications from 2009 onward, as earlier research on the topic was scarce and fragmented, preventing a meaningful synthesis of the evidence. The literature search was conducted on three databases: PubMed, Scopus, and Web of Science. The databases were searched using keywords related to “rhythm” and “language”. Specifically, this string was entered: (rhythm* OR tempo OR entrainment) AND (training OR intervention OR treatment OR rehabilitation OR potentiat* OR enhanc*) AND (child* OR development) AND (language OR speech OR phonolog* OR reading OR dyslexia) NOT (EEG OR heart OR stuttering OR cochlea* OR danc* OR Parkinson OR circadian). The search terms were applied to the title, abstract, and keywords fields and, where possible, limited to research articles and reviews. Additionally, the reference lists of the resulting records were examined to identify other relevant studies.

Studies meeting the following criteria were excluded: (i) studies conducted with participants over the age of 18; (ii) studies that did not involve training protocols; (iii) studies involving a single case or fewer than 10 participants per group; (iv) studies using music training that did not include a clear rhythmic component (i.e., studies that mentioned only “music” or lacked sufficient detail about the rhythmic aspects were excluded); (v) studies that lacked a description of the procedure; (vi) studies lacking a control group or control training; (vii) non–peer-reviewed publications (i.e., dissertations, theses, or conference proceedings); (viii) studies that did not report clear linguistic outcome measures.

### Selection process and screening

**Fig. 1:**
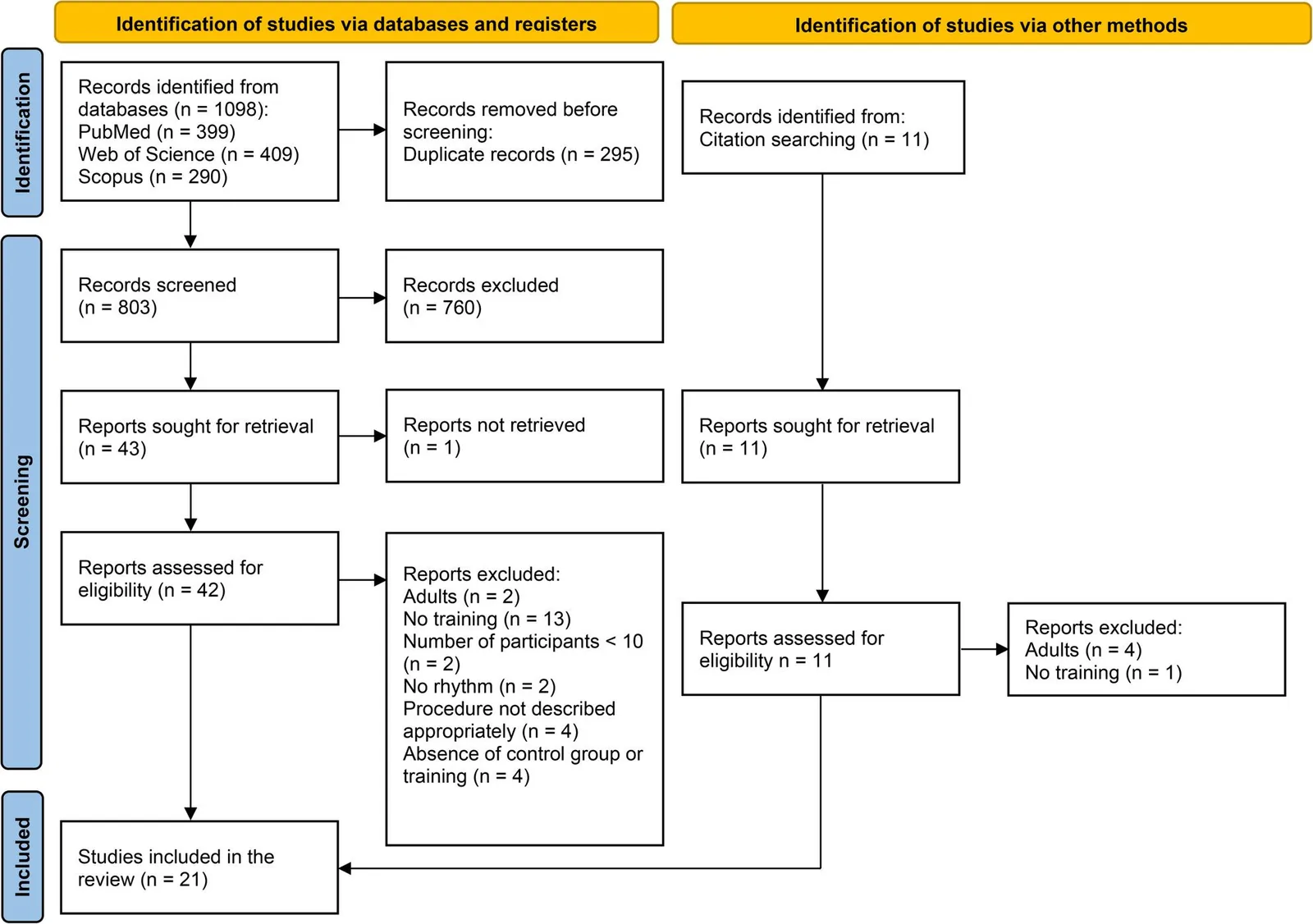
The flowchart depicts the systematic literature search and study selection process following PRISMA guidelines [45]

Figure 1 shows the flow chart of the study selection process. Initial search identified 1098 potential articles. After removing 295 duplicates, 803 remained for screening. This process was completed by two authors (C.D.P. and V.T.) who independently worked on Rayyan [46], an online platform for systematic reviews. Disagreements were resolved by consultation with a third co-author. In the initial step, manuscripts were excluded if their titles and abstracts were clearly inappropriate. Out of the 803 articles screened, 42 met the inclusion criteria and were subjected to a full-text examination. Eleven additional articles were identified from the reference lists.

### Quality check

To evaluate the quality of each included study, we developed two ad hoc checklists inspired by the Critical Appraisal Skills Programme Randomised Controlled Trial Checklist (CASP [47]): one aimed at studies implementing multiple-session protocols conducted in real-world settings (i.e., school or clinical facility), and the other at studies implementing single-session protocols mostly conducted in laboratory settings. The assessment was conducted independently by two authors (C.D.P. and V.T.), based on the information provided in the main text and supplementary materials. Particular attention was given to group differences at baseline and in pre-post changes. All selected studies received a score of 65 or higher, suggesting that they met a minimum threshold for quality. However, all of them have methodological limitations that need to be taken into account. The full results of the quality assessment are presented in Supplementary Table S1. All training studies reported in Table S1a had a between-subjects design and used a pseudo- or cluster-randomization procedure to balance baseline characteristics across groups (e.g., age, reading level, IQ). In contrast, all training studies reported in Table S1b employed a within-subjects design and counterbalanced the training order. These studies have higher internal validity compared to real-world intervention studies but exhibit some methodological limitations, such as the lack of pre-training measurements (see Discussion) and lower ecological validity.

## Results

A total of 21 articles were included in the review according to the considered criteria. For each article, the following information was extracted: participants’ characteristics, training’s characteristics, type of setting (individual or group), control training’s characteristics, number and duration of sessions, linguistic outcome measures, and statistically significant results. Follow-up studies were discussed separately due to their limited number (k = 3) and inconsistent follow-up timing.

We classified the studies into three categories based on the type of rhythmic training: (1) studies using musical training, which includes musical activities based on rhythmic patterns (music-based rhythmic training; k = 9); (2) studies using rhythmic patterns combined with linguistic material, such as phoneme and words (language-based rhythmic training; k = 5); and (3) studies using rhythmic patterns that prime linguistic tasks (rhythmic priming sequences; k = 7). All relevant information for each study is summarized in Tables 1 and 2, and 3.

### Participants’ characteristics

All the studies involved children or pre-adolescents aged between 5 and 12 years. They could include: (1) typically developing children (TD), characterized by age-appropriate language and cognitive skills; (2) children with developmental language or speech disorders (DLD), who show deficits in language comprehension and/or production that cannot be attributed to peripheral deficits or global impairments in other cognitive domains [48]; and (3) children with reading disorders, including developmental dyslexia (DD) that affects the visual processing of words while leaving oral and non-verbal reasoning skills intact [49].

Seven studies using music-based rhythmic training recruited TD participants only [50–56]. Two studies targeted clinical samples: one recruited poor readers [57] while the other included children with DD [58].

Almost all studies adopting language-based rhythmic training involved children with DD [59–62], with the exception of one that recruited TD children [63].

All studies employing rhythmic priming sequences included TD children. Moreover, four of these also compared the effects of rhythmic priming between TD children and children [28 Exp 1a, 64–66] with DLD or with DD [28 Exp 1b].

### Study design

As required by the inclusion criteria, all the studies included a control group and/or a control training condition. Specifically, all music-based rhythmic programs and all language-based rhythmic protocols adopted a mixed group **×** time (pre-post) design, in which the experimental group was assigned to rhythmic training, while the control group was assigned to control training. Both groups underwent linguistic tasks before and after the training to assess pre-post differences in performance. In some studies [55, 60, 62], the control group was assigned to a passive condition (i.e., no training). All other studies used an active training condition (e.g., painting or sports activities).

Studies using rhythmic priming sequences adopted a within-subjects design. In these cases, participants were exposed to either a regular rhythm (experimental condition) or an irregular rhythm (control condition) and were subsequently assessed on linguistic tasks.

### Type of setting

A total of 12 studies [28, 55, 57, 59–62, 64–68] employed individual training settings, while 9 studies [50–54, 56, 58, 63, 69] implemented group-based training. Both individual and group formats appear to be effective in rhythm-based intervention.

### Outcome measures

The majority of studies examined phonological awareness measures (such as phonemic segmentation of words, phonemic blending, and rhyme detection) [50, 52, 53, 57–60, 63] and reading fluency (mainly speed and accuracy in words, pseudo-words, and text reading) [54–63]. Sometimes, the rapid automatized naming (RAN), speech segmentation, and pseudo-word reproduction were assessed as well [51, 57, 59, 60]. In rhythmic priming studies participants were required to perform grammaticality judgement tasks on sentences, except Fiveash et al. [65], in which participants were required to perform a sentence repetition task.

In some studies, the effect of training on language-related, non-literacy outcome measures has also been examined. Specifically, studies using music-based or language-based training assessed pre-post differences in rhythmic abilities, with tasks involving rhythm discrimination, reproduction, and synchronization, and temporal processing abilities, with rise time and temporal anisochrony discrimination tasks. In some cases, cognitive tests, assessing auditory and visual attention, visuospatial abilities, and interference control abilities, were also added.

### Results – Music-based rhythmic training

The music-based rhythmic protocols exposed children to rhythmic music, either generated by pre-recorded audio tracks or played live on instruments, and required them to discriminate or synchronize with the rhythm. To this end, children participated in various activities, such as music reproduction, drumming, and singing. These protocols were founded on well-established music education methodologies, including the approaches of Kodaly, Nykrin, Orff, and Wuytack which integrate structured musical learning, including theory, singing and notation (Kodaly), with creative, experimental activities that encourage movement, improvisation and active engagement (Nykrin, Orff, and Wuytack). Most of the musical activities were collaborative (e.g., joint drumming, singing, or clapping) and conducted in group settings (7 studies out of 9 studies; see Table 1). In all cases, the training involved movements. In some cases, linguistic materials were also incorporated [53, 57, 58]. For example, children were asked to identify the rhythm of a poem or replace syllables of words with sounds.

**Table 1:** Studies using music-based rhythmic training

Study	Participants	Music-based rhythmic training	Linguistic	Setting	Control training	Number of sessions	Outcomes measures	Results
Typical Development (TD)								
Degé & Schwarzer (2011) [50]	EG: *n* = 13 TD (age: M = 5.9 y, SD = 3.9 m); CG(a): *n* = 14 TD (age: M = 5.7 y, SD = 4.2 m); CG(b): *n* = 14 TD (age: M = 5.2 y, SD = 4 m)	Training based on a program for early music education that included joint singing, joint drumming, rhythmic exercises, meter execution, training of rudimentary notation skills, dancing, and playful familiarization with intervals.	No	G	CG(a): Phonological training including listening, rhyming, and phoneme recognition exercises. CG(b): Sport training including exercises to train balance, physical strength, endurance, coordination, fine-motor abilities, body perception and relaxation.	10 min per session, for 20 weeks, for a total of 100 sessions.	PA Detection of rhymes, segmentation of words into syllables, synthesis of phonemes into words, and phoneme recognition in words.	Significant time × group interaction: only the EG and the CG(a) groups showed pre-post improvement in the overall aggregate score and in the aggregate score for large units (words, i.e., the first two subtests). All groups showed pre-post improvement in the aggregate score for small units (phonemes, i.e., the last two subtests).
Francois et al. (2013) [51]	*n* = 24 TD (age: M = 8 y, SD = 0.4) pseudorandomly divided into two groups.	Training based on a program for music education.	Not reported	G	Painting training.	45-min session, twice a week for 1 year.	Speech segmentation.	Only the EG showed a pre-post improvement.
Frey et al. (2022) [52]	EG: *n* = 42 TD (age: M = 5.9 y, SD = 3.8 m); CG: *n* = 34 TD (age: M = 5.8 y, SD = 4.3 m)	Training based on a program for music education that included four activities: repeating rhythmic sequences using one or more body parts; repeating rhythmic sequences with different body parts while synchronizing with instrumental music of various styles, tempo, and metrics; moving around the classroom in sync with various soundtracks; and playing a game that involved either walking to the tempo of the music or creating movements to match the rhythm. Activities could be supported by videos.	No	G	Art training, which included visuo-spatial and motor activities, such as copying or miming artworks (either statically or through movement); acting out an emotion, character, or object from an artwork; guessing mimed objects or actions related to an object.	5 min per day, 5 days per week, over a 10-week period.	PA Syllable counting, syllable deletion, rhyme discrimination, phoneme identification, invented spelling.	Both groups did not improve after training.
Moreno et al. (2009) [56]	EG: *n* = 16 TD (age: M = 8.4 y, SD 4.3 m); CG: *n* = 16 TD (age: M = 8.2 y, SD = 4.7 m)	Training based on a program for music education on rhythm, melody, harmony and timbre that included production and improvisation of rhythms in different tempi and meters; production and improvisation of melodies; recognition, discrimination and production of harmonic progression; recognition of timbres from different instruments and voices.	No	G	Painting training, which emphasized the recognition and application of light and color, line and perspective, as well as materials and textures.	One 75-min session, twice a week for 24 weeks.	R Word reading (errors).	Only the EG group improved significantly after the training.
Patscheke et al., 2019 [53]	EG(a): *n* = 13 TD (age: M = 5.6 y, SD = 0.5); CG(a): *n* = 13 TD (age: M = 5.2 y, SD = 0.7); CG(b): *n* = 14 TD (age: M = 5.6 y, SD = 0.4)	Training based on a program for early music education that included exercises of joint drumming using various percussion instruments and meter execution through body movements synchronized to a beat; perception and imitation of rhythmic sounds, such as nursery rhymes accompanied by clapping or complex rhythmic sequences combined with spoken syllables. No singing or musical accompaniment was included.	Yes	G	CG(a): Pitch training, which included joint singing of songs and rhymes; intonation exercises (reproducing tone sequences and intervals); pitch discrimination; and listening exercises focused on identifying animal calls and musical instruments. CG(b): Sports program focused on motor skills and body coordination, including training in balance, physical strength, endurance, body awareness, and relaxation techniques.	20 min, three times a week for 16 weeks.	PA Segmentation of words into syllables, detection and production of rhymes, synthesis of onset phonemes and words, and onset phonemes recognition in words.	All groups showed improvement in the aggregate score after the training. No significant time × group interaction was found. When comparing pairs of groups, the CG(a) demonstrated a greater improvement in phonological awareness at the word level compared to the CG(b).
Rautenberg (2013) [54]	EG: *n* = 33 TD (age: M = 7.8 y, SD = 0.4); CG(a): *n* = 41 TD (age: M = 7.7 y, SD = 0.4); CG(b): *n* = 85 TD (age: M = 7.1 y, SD = 0.4)	The training aimed to enhance rhythmic and tonal/melodic skills, as well as auditory discrimination of timbre and sound intensity. It included various activities, such as discriminating between rhythmic patterns and reproducing them using instruments or body movements; identifying tone pitches and melodies; responding to different meters or tonalities through movement; recognizing various forms of notation and learning to use these symbols by playing rhythmic or melodic instruments.	No	G	CG(a): Visual art training, which sensitized the children to different forms, colors and materials that can be used for printing. CG(b): No training.	Twice a week for 8 months.	R Word reading (accuracy, speed, prosody).	Higher performance in reading accuracy in EG compared to CGs. All groups improved in reading speed.
Zanto et al. (2024) [55]	EG: *n* = 32 TD (age: M = 8.9 y); CG: *n* = 21 TD (age: M = 8.9 y)	The training was conducted using a custom video game called “Coherence”, where participants tapped the tablet screen in sync with a visual cue representing the musical beat. The difficulty adapted along three dimensions: temporal complexity, spatial complexity, and memory load, starting at the lowest levels for each song. A total of 16 difficulty levels were available.	No	I	No training.	20-min sessions, three times per week for 6 weeks.	R Reading fluency.	Significant time × group interaction: the EG not the CG exhibited improved performance.
Poor readers								
Bhide et al. (2013) [57]	EG: *n* = 10 poor readers (age: M = 6.9 y, SD = 0.3); CG: *n* = 9 poor readers (age: M = 6.7 y, SD = 0.4)	The training included various activities: tapping a space bar at the same time as a metronome; discriminating metronome tempos or short rhythms; discriminating sound rise time durations; clapping and marching to the beat of a song or other hand-clap games; learning to chant; recognizing the rhythm of a poem; playing a game in which syllables of words are replaced with sounds, which reproduce their stress pattern, and words have to be detected based on their prosody.	Yes	I	Reading training: children hear sounds, rhymes, and words and have to match them to correct spelling or make decisions about whether words rhyme.	19 sessions in 2 months, 4 or 5 activities per session.	PA, N, R Word and pseudo-word reading, spelling, oddity rhyme, phoneme deletion, RAN.	The EG improved in all measures except RAN, while the CG improved in all measures except the oddity rhyme test. When considering an aggregate measure, the effect of group and the time × group interaction were both non-significant.
Developmental dyslexia (DD)								
Flaugnacco et al. (2015) [58]	EG: *n* = 24 DD; CG: *n* = 24 DD (age: M = 10 y, SD = 1)	Training based on a program for music education that included the use of percussive instruments, rhythm based on syllables, rhythmic body movements accompanying music, sensorimotor synchronization games.	Yes	G	Painting training that emphasized visual-spatial and hand skills as well as creativity. Both the EG and CG group received a “conventional” training for reading, carried out daily at home for 20 min for seven months, with parents’ supervision.	2 h/week over a period of 7 months	PA, R Word, pseudo-word and text reading, pseudo-word reproduction, phonemic segmentation, phonemic blending.	Both groups improved in reading accuracy (pseudo-words and text reading, pseudo-word reproduction, phonemic segmentation, phonemic blending) and speed (word, pseudo-word, and text reading). Larger improvement was found in the EG in accuracy (pseudo-words and text reading, pseudo-word reproduction, phonemic blending).

*Y* years, *m*, months, *M* mean, *SD* standard deviation, *EG* experimental group, *CG* control group, *TD* typical development, *DD* developmental dyslexia,* Linguistic* the training includes linguistic materials, *I* individual, *G* group, *PA* phonological awareness tasks, *R* reading tasks, *N* naming, *RAN* rapid automatized naming

The training assigned to the control group consisted of non-linguistic activities (e.g., sport, painting, art) or in programs designed to enhance phonological awareness, in TD preschool children [50] or poor readers [57]. In the study by Flaugnacco et al. [58], all participants were children with DD who received a conventional reading training together with a rhythmic or a painting training. Notably, in the study by Patscheke et al. [53], a musical control condition was implemented, which focused on training the melodic instead of the rhythmic component of music.

When the control training consisted of phonological exercises aimed at enhancing reading or pre-literacy skills, both the experimental and control groups showed significant improvements of comparable magnitude, in both TD children [50] and poor readers [57]. In these cases, the effect of rhythmic training and phonological training protocols was medium to large in size. By contrast, when the control training involved visual art activities, such as painting, only the experimental group showed improvement, as demonstrated by studies involving TD children, either in reading accuracy [54, 56] or in speech segmentation [51]. Similarly, when children with DD received conventional reading training combined with either a music-based rhythmic training or a painting training, both the experimental and control groups showed improvements in reading accuracy and speed, but the former showed a significantly greater improvement in reading accuracy [58].

In some cases, improvements in phonological awareness at post-training were also observed in the control group when the training involved sports activities only, targeting, for example, coordination, fine motor skills, and body perception, as reported in a study with TD children [50].

Only one study out of nine [52], conducted with TD children, did not show improvements in linguistic abilities following either rhythmic or control training. Nonetheless, in this study, a positive correlation was found at pre-training between individual rhythmic abilities (rhythm discrimination and synchronization) and phonological awareness (syllable counting and phoneme identification). The authors attributed the null result to the short duration of the intervention and emphasized the importance of sufficient training exposure to produce measurable linguistic or cognitive gains. In this regard, we found that the mean total training duration across studies was approximately 30 h (ranging from 1.7 to 78 h), with a mean frequency of 2 to 3 sessions per week (ranging from 1 to 5 sessions per week).

#### Non-literacy measures

In addition to reading skills, phonological awareness, and naming abilities, Bhide et al. [57] investigated whether the effects of musical training and the accompanying reading training transferred to children’s verbal working memory and their ability to detect onset speed of sounds (i.e., the perception of the rise time in the amplitude envelope of a sound). To this end, the authors examined performance on the verbal digit span test (both forward and backward) in a sample of poor readers and measured rise time discrimination thresholds. These abilities are known to be associated with rhythmic processing and are often impaired in children with DD or phonological deficits [70, 71]. In particular, children with DD tend to have difficulty in discriminating prolonged rise times of sounds, rather than their frequency or intensity [72]. Digit span performance significantly improved and rise time discrimination thresholds significantly decreased selectively in the group who underwent rhythmic training. Higher digit span scores following rhythmic training were also reported in DD [58] and TD [56] children. Conversely, children who received painting training outperformed those in the rhythmic group on the Block Design subtest of the Wechsler Intelligence Scale for Children.

Flaugnacco et al. [58] further examined the effects of music-based rhythmic training on several measures, including auditory attention, rise time and temporal anisochrony discrimination, rhythm reproduction, metrical perception, and tapping. While both the rhythmic training and control groups improved on most measures, except metrical perception, the rhythmic group demonstrated significantly higher gains in auditory attention, temporal anisochrony discrimination, and rhythm reproduction. Similarly, Frey et al. [52, 55] reported improvements in sensorimotor synchronization following rhythmic training compared to an art training group in TD children, in line with the study by Zanto et al. [55]. Moreover, Zanto et al. [55] found that both experimental and passive control groups improved in visuo-spatial working memory (Corsi block-tapping task), as well as in verbal (Stroop task) and non-verbal (the Flanker task) interference control, although the experimental group demonstrated greater gains in the latter task.

Table 1 Characteristics of the studies using *rhythm-based musical training*. The presence of linguistic material is represented in the fourth column. Only statistically significant results are reported.

### Results – Language-based rhythmic training

A group of four studies investigated the efficacy of combining verbal material (e.g., syllables, words, pseudo-words, phrases) with rhythmic accompaniment during reading exercises [59–62]. This type of program was named “Rhythmic Reading Training” (RRT). In these studies, regular rhythms were generated either by computer-played audio tracks of metronomic beats or by simple sequences performed using percussion instruments (e.g., wooden sticks) or clapping. In one study [61], reading exercises were alternated with synchronization games that involved small musical instruments, such as bongo, maracas, and xylophone. In another study [60], rhythms were accompanied by visual stimuli, for example, visual targets where words changed color in synchrony with each beat or images. All these studies involved children with DD, with active control groups engaged in alternative reading training [59, 61].

In this category of studies, we included one involving TD children, in which regular rhythms were generated by verbal materials themselves through syllabic manipulations [63]. In an example of activity, children listened to a story with an altered endings that broke the rhymes but preserved the rhythm (“Slinky Malinki was blacker than black, a stalking and lurking.”) and had to choose the final word (“adventurous cat”, “scary cat”, or “misbehaving cat”), thus fostering rhythmic and phonological awareness. The other studies using language-based rhythmic training involved children with DD (see Table 2) and compared a group exposed to rhythmic training (experimental group) with a group receiving a specific reading intervention for dyslexia [59, 61], a math training [63], or a passive control condition [60, 62].

**Table 2:** Studies using language-based rhythmic training

Study	Participants	Language-based rhythmic training	Setting	Control training	Number of sessions	Outcome measures	Results
Typical Development (TD)							
Harrison et al. (2018) [63]	EG: *n* = 27 TD; CG(a): *n* = 24 TD; CG(b): *n* = 19 TD (age: M = 4 y 6 m, range = 4 y 1 m − 5 y)	The children were exposed to audio stimuli and trained in discriminating intonation, and timing of words or sentences. The training also included a group activity in which children had to complete a story with a strong and predictable rhythm.	I and G	CG(a): Phonological awareness training, which included rhyming games, letter and sound identification (taken from “Sound Linkage”; Hatcher, 2000). CG(b): Math training, which included number recognition, matching colors, matching shapes, simple addition and subtraction, domino games.	10 weekly sessions of 15 min each.	PA, R Word reading, phonological awareness tasks (rhyme detection and alliteration detection), speech-rhythm sensitivity tasks (stress, intonation, and timing).	Higher pre-post difference in word reading accuracy in the EG compared to the CG(b), but not compared to the CG(a). Higher pre-post difference in speech-rhythm sensitivity (intonation and stress) in the EG compared to the CG(b), but not compared to the CG(a). No differences between the two CGs. Pre-post differences were controlled for pre-training scores, vocabulary, and age.
Developmental dyslexia (DD)							
Bonacina et al. (2015) [62]	EG: *n* = 14 DD; CG: *n* = 14 DD (age: M = 12.1 y, SD = 1.1)	The “Rhythmic Reading Training” (RRT) included reading syllables, words, pseudo-words, and phrases presented on a computer screen in synchrony with a rhythmic accompaniment, which could be either a regular metronomic beat or a simple pentatonic melody played by drums, wooden sticks, or hand clapping.	I	No training.	9 biweekly sessions, 30 min each. Each session was preceded by one training session.	R Word, pseudo-word, and text reading.	Both groups improved in low-frequency word reading accuracy and low-frequency long word reading speed. In addition, the EG improved in pseudo-word reading speed and text reading accuracy
Cancer et al. (2020) [59]	EG: *n* = 12 DD (age: M = 9.7 y, SD = 1.4); CG: *n* = 12 DD (age: M = 9.9 y, SD = 1.9)	RRT (as Bonancina et al., 2015).	I	A treatment for dyslexia that combines the Visual Hemisphere-Specific Stimulation (Bakker and Vinke, 1985; Lorusso et al., 2006), which consists of the lateralized tachistoscopic presentation of words, and the Action Video Game Training (Franceschini et al., 2013), which consists of playing video games that capitalizes speed, visual-attention, and motor abilities.	45 min, three times per week, for 3 weeks.	PA, N, R Word, pseudo-word, and text reading, phonemic blending and phoneme elision (phonological awareness), RAN.	Both groups improved in word and pseudo-word reading speed. In addition, the EG improved in phonemic blending, whereas the CG improved in pseudo-word reading accuracy and text reading speed.
Cancer et al. (2022) [60]	EG(a): *n* = 21 DD (age: M = 10.7 y, SD = 1.7); EG(b): *n* = 21 DD (age: M = 10.6 y, SD = 1.4); CG: *n* = 16 DD (age: M = 11 y, SD = 1.9)	EG(a): RRT with visual cues synchronized with the auditory rhythmic stimulation (i.e., the words changed color simultaneously to each beat). EG(b): standard (only auditory) RRT.	I	No training.	10 sessions of 45 min over 5 weeks.	PA, N, R Word, pseudo-word, and text reading, phonemic elision, RAN.	Only the two EGs improved in word, pseudo-word, and text reading speed, pseudo-word reading accuracy, phonemic elision, and RAN.
Cancer et al. (2023) [61]	EG: *n* = 16 DD; CG: *n* = 16 DD (from 4th to 8th grade)	RRT alternated with musical games involving synchronization activities with small instruments (e.g. bongo drums, maracas, xylophone).	I	Reading training: activities focused on reading speed and accuracy, reading-related skills, and metacognitive activities. Both paper and pencil and computer-based activities were used.	20 sessions of 60 min each, twice a week.	R Word and pseudo-word reading, text reading.	Both groups improved in reading speed (words, pseudo-words, and text). In addition, the CG improved in reading accuracy (words, pseudo-words, and text).

*Y* years, *m* months, *M* mean, *SD* standard deviation, *EG* experimental group, *CG* control group, *TD* typical development *DD* developmental dyslexia *RRT* rhythmic reading training, *I* individual *G* group, *PA* phonological awareness tasks, *R* reading tasks, *N* naming, *RAN* rapid automatized naming

The results of these studies showed that both the group of children with DD who underwent the RRT and the group of children with DD who received reading intervention improved in reading speed after training [59, 61]. In addition, the experimental group improved in phonological awareness (i.e., phonemic blending task [59]) and reading accuracy [61]. The addition of musical synchronization games to the RRT did not lead to further gains in reading performance [61].

When the control group participated in a program combining tachistoscopic word presentation (350 ms) with action video games, improvements were observed in pseudoword accuracy and text reading speed. When children with DD were compared to a passive control group that did not receive any intervention, both groups showed improvement in low-frequency word reading. However, only the experimental group improved in pseudo-word reading speed and text reading accuracy [59], or, in another study, only the experimental group showed improvements across all outcome measures, including word, pseudo-word, and text reading speed, pseudo-word reading accuracy, phonemic elision, and RAN [60, 63].

Interestingly, Harrison et al. [63] found that in TD children, when outcome measures were controlled for pre-training scores, vocabulary, and age, differences between speech-rhythm training and phonological awareness training were not significant. Notably, only the experimental group showed a pre-post improvement that was statistically greater than that of the control group undergoing math training, while the group exposed to phonological training did not.

The mean duration of the total training was approximately 8 h (ranging from 2.5 to 20 h), with a mean frequency of 1–2 sessions per week (ranging from 1 to 3 sessions per week).

#### Non-literacy measures

Results on language-related outcomes, all measured in groups of children with DD, are mixed. In Bonacina et al. [62], both the experimental and the passive control group showed improvement in a rhythm reproduction task after training, indicating a specific training effect on rhythm abilities. In Cancer et al. [59], however, neither the experimental nor the control group improved in rhythm discrimination, rhythm reproduction, or auditory attention tasks after training. In contrast, Cancer et al. [60] found that language-based rhythmic programs, with either visual or standard auditory cues, produced significant improvements in visual attention, rhythm reproduction, and synchronous tapping abilities.

Table 2 Characteristics of the studies using *language-based rhythmic training*. Only statistically significant results are reported.

### Results – Rhythmic priming sequences

Seven studies have examined the efficacy of rhythmic priming sequences delivered in a single training session [28, 64–69]. In these studies, participants were first exposed to regular rhythmic sequences produced by percussion instruments (recorded tracks), such as tam-tam, maracas, followed shortly after by a linguistic task (see Table 3).

**Table 3:** Studies using rhythmic priming sequences

Study	Participants	Rhythmic priming sequences	Control training	Number of sessions	Outcome measures	Results
Typical development (TD)						
Canette et al. (2020) [67]	TD: *n* = 16 (age: M = 8 y, SD = 6 m)	Regular rhythmic priming sequences of 17 s played by percussion instruments (e.g., cymbal, drum, snare drum, bass drum).	Irregular rhythmic musical priming sequences of 17 s (textural sounds).	One session of 12 blocks: 6 starting with the regular priming sequence and 6 with the irregular priming sequence, in a counterbalanced order.	At the end of each sequence, a grammaticality judgment task on sentences was performed (“syntax task”).	Higher performance after the regular compared to the irregular primes.
Chern et al. (2018) [68]	TD: *n* = 16 (age: M = 6.5 y, SD = 11 m)	Regular rhythmic priming sequences of 32 s played by two percussion instruments (tam-tam and maracas), as in Przybylski et al. (2013).	Irregular rhythmic musical priming sequences of 32 s, with the same number of tones as the regular sequences.	One session consisting of 6 blocks: 3 starting with the regular prime and 3 with the irregular prime, in a pseudorandom order.	At the end of each sequence, a grammaticality judgment task on sentences was performed.	Higher performance after the regular compared to the irregular primes.
Fiveash et al. (2020) (Exp 1) [69]	TD: *n* = 32 (age: M = 8.7 y, SD = 6.2 m)	Regular rhythmic priming sequences of either 8–16 s played by percussion instruments (e.g., cymbal, drum, snare drum, bass drum).	Irregular rhythmic musical priming sequences (textural sounds) with the same acoustic information as the regular sequences (event duration, total duration, and instruments).	One session of 16 blocks: 8 short and 8 long, 8 regular and 8 irregular, in a pseudorandom order.	At the end of each sequence, grammaticality judgment task on sentences was performed.	No benefit of the regular over the irregular primes at either 8–16 s durations.
Fiveash et al. (2020) (Exp 2) [69]	TD: *n* = 36 (age: M = 8.6 y, SD = 5.6 m)	As Exp 1, two sequences of 16-s and four sequences of 32-s.	As Exp 1.	As Exp 1.	At the end of each sequence, a grammaticality judgment task on sentences was performed.	Higher performance after the regular compared to the irregular primes after the 32-s sequences. No differences after the 16-s sequences.
Developmental language disorder (DLD) and typical development (TD)						
Bedoin et al. (2016) [64]	DLD: *n* = 16 (age: M = 9.6 y, SD = 13 m); TD: *n* = 16 (mean age = 9.2 y, SD = 17 m)	Regular rhythmic priming sequences of 32 s played by two percussion instruments (tam-tam and maracas), as in Przybylski et al. (2013).	Non-rhythmic priming sequences consisting of environmental sound scenes of 30 s.	One session of 8 blocks: 4 starting with the regular prime and 4 with the irregular prime, in a pseudorandom order.	At the end of each sequence, a grammaticality judgment task on sentences was performed.	Higher performance after the regular compared to the irregular primes in both groups. No significant interaction.
Fiveash et al. (2023) [65]	TD: *n* = 18 TD (age: M = 8.8 y, SD = 23.3 m); DLD: *n* = 15 (age: M = 9.2 y, SD = 28.3 m)	Regular rhythmic priming sequences of 32 s. selected from Fiveash et al. (2020).	Irregular rhythmic musical priming sequences of 32 s containing the same notes and durations as the regular sequences.	One session of 6 blocks: 3 starting with the regular prime and 3 with irregular prime, in a counterbalanced order.	At the end of each sequence, the children performed a sentencerepetition task.	Higher performance after the regular compared to the irregular primes in both groups. When reading age was entered as covariate, the pre-post improvement after the regular compared to the irregular prime survived in the TD only.
Ladányi et al. (2021) [66]	DLD: *n* = 17 (age: M = 6.2 y, SD = 6 m); TD: *n* = 17 (age: M = 6.2 y, SD = 6 m)	Regular rhythmic priming sequences of 32 s played by two percussion instruments (tam-tam and maracas), as in Przybylski et al. (2013).	(a) Irregular rhythmic musical priming sequences of 32 s, with the same number of tones as the regular sequences. (b) “Baseline” condition (silence).	One session of 6 blocks: 2 starting with the regular prime, 2 with the irregular prime, and 2 with the silence, in a pseudorandom order.	At the end of each sequence, a grammaticality judgment task on sentences and a picture-naming task were performed.	Higher performance after the regular prime compared to the irregular prime and to baseline, in both groups. No significant difference between the irregular prime and the baseline and no significant interaction with the group.
Przybylski et al. (2013) (Exp 1a) [28]	DLD: *n* = 12 (age: M = 9.5 y, SD = 23 m); TD: *n* = 20 (age: M = 9.4 y, SD = 20 m)	Regular rhythmic priming sequences of 32 s played by two percussion instruments (tam-tam and maracas).	Irregular musical priming sequence of 32 s played by two percussion instruments.	One session of 8 blocks: 4 starting with the regular prime and 4 with irregular prime, in a pseudorandom order.	At the end of each sequence, a grammaticality judgment task on sentences was performed.	Higher performance after the regular compared to the irregular prime, in both groups. No significant interaction.
Developmental dyslexia (DD) and typical development (TD)						
Przybylski et al. (2013) (Exp 1b) [28]	DD: *n* = 10 (age: M = 9.7 y, SD = 10 m); TD: *n* = 18 (age: M = 9.8 y, SD = 10 m)	As Exp 1a.	As Exp 1a.	As Exp 1a.	As Exp 1a.	Higher performance after the regular compared to the irregular prime, in both groups. No significant interaction.

*Y* years, *m* months, *M* mean, *SD* standard deviation, *EG* experimental group, *CG* control group,* TD* typical development, *DD* developmental dyslexia, *DLD* developmental language disorder

The control condition consisted in the exposure to irregular rhythmic priming sequences of the same duration as the regular sequences. These control sequences were musical, produced by the same percussion instruments, and matched the regular sequences in acoustic characteristics. In one study [64], the control sequences consisted of environmental sound scenes without temporal regularity, while in others they were textural sounds [67] with the same acoustic information as the regular rhythmic sounds [69]. Ladányi et al. [66] added a third, silent control condition/group. Notably, none of the rhythmic priming sequence protocols involved linguistic stimuli or motor activities.

Improved performance in grammaticality judgment tasks was found after exposure to either 32-second [28, 64, 65, 68, 69 Exp 2] or 17-second [67] regular rhythmic priming sequences, compared to irregular priming sequences. Similarly, better performance in sentence repetition was observed after exposure to a 32-second regular prime compared to an irregular prime in TD children [65]. In contrast, no benefit of regular over irregular priming was observed for shorter exposures of 8 and 16 s [69]. Interestingly, improvements following exposure to regular rhythms were seen not only in TD children [67, 68, 69 Exp 2] but also in children with DLD [28 Exp 1a, 64, 66] and DD [28, 34 Exp 1b].

#### Non-literacy measures

No differences between regular and irregular rhythmic sequences emerged when these preceded a visuo-spatial attention task (a cancellation task adapted from the Wechsler Intelligence Scale for Children [65, 68]), a math task [68], or a non-verbal Stroop task [66], regardless of the group (TD or DLD).

Table 3 Characteristics of the studies using *rhythmic priming sequences*. Only statistically significant results are reported.

## Discussion

This literature review aims to provide an overview of existing evidence on the effects of rhythmic training on language and language-related functions in childhood. Among the twenty-one selected studies, three main categories of training were found: music-based rhythmic training, language-based rhythmic training, and training with rhythmic priming sequences. All studies utilizing music-based rhythmic training, except one [52], demonstrated greater improvements in the experimental groups compared to control groups trained in visual art [51, 54, 56, 58], sport [50, 53], or receiving no training [55]. Specifically, their gains concerned phonological processing, such as speech segmentation [51] and phonological awareness [50, 53, 58], as well as reading accuracy [54, 56, 58] and fluency [55].

Notably, when contrasted with control groups undergoing phonological awareness or reading training [50, 53, 57], improvements were comparable, suggesting that both approaches may have engaged shared neurocognitive mechanisms [32, 73]. Moreover, the benefits of music-based rhythmic training were independent of the melodic component [74], as clearly demonstrated by the study of Patscheke et al. [53], which isolated rhythm from melody and still observed positive effects, and by the study of Rautenberg et al. [54], which found that reading accuracy in young children correlated with rhythmic timing sensitivity, but not pitch sensitivity. Likewise, the benefits of rhythmic priming sequences (Table 3), which did not involve the melodic component, provided additional evidence of the superiority of regular over irregular sequences.

The music-based rhythmic training protocols were drawn from validated educational programs and included a variety of activities such as clapping, dancing, and singing, mainly involving tempo recognition and reproduction. Some of these activities required the synchronization of body movements. The finding that rhythmic training produced significantly greater improvements in phonological processing compared to motor training alone (sport activities) suggests that movement per se does not fully explain gains in language functions. On the other hand, in the studies by Degé and Schwarzer [50] and Patscheke et al. [53], children who participated in sports training also demonstrated improvements in certain phonological awareness tasks. This effect may be attributed to shared underlying components between motor and rhythmic skills, such as coordination and timing, which partially overlap with mechanisms supporting language processing.

Language-based rhythmic protocols focused on synchronizing verbal material (e.g., syllables, words) with rhythmic patterns, and were primarily designed for children with dyslexia. These interventions leveraged the integration of linguistic activities (e.g., reading) with rhythmic structures. All these training programs obtained improvements in phonological awareness [59, 60], rapid phonological/lexical access [60], as well as reading accuracy and speed [59–63]. Notably, children who received training based on rhythms derived from verbal metrics showed gains in word reading accuracy comparable to those achieved through phonological awareness training [63]. Consistent results emerged in a study by Thomson et al. [75], in which children received a rhythmic or a phonemic segmentation training program and both groups showed improvements of a similar extent (i.e., medium to large effect size). In some cases, the outcomes were superior to those observed in children receiving validated programs for dyslexia [59]. Interestingly, the rhythmic training employed by Harrison et al. [63] emphasized prosodic elements, such as stress, intonation, and timing, and found results in reading comparable to those observed with a phonological awareness training.

The main limitation of language-based rhythmic programs, compared to the other protocols, is that when rhythm and language are presented concurrently their specific contributions become difficult to disentangle. Moreover, the generalizability of these results is limited by sample characteristics, as most studies have focused on children with DD.

From a methodological point of view, studies employing rhythmic priming sequences offer a valuable advantage. They allow for direct comparisons between regular and irregular rhythmic conditions within the same participant, and include TD, as well as DLD and DD children. All studies using training with rhythmic priming found significant improvements in grammatical judgment tasks (i.e., syntactic processing) after exposure to regular rather than irregular sequences, and this evidence emerged in children with typical or atypical development. The design of these studies reinforces the notion that temporal regularity, rather than other musical or movement-related features, is the primary driver of linguistic enhancement. The rhythmic priming sequences appeared to require a minimum exposure duration of 17 s to be effective. As for the language-based protocols, the priming sequences provide evidence for the efficacy of rhythmic training on linguistic skills without the confounding influence of melody and motor components.

Unfortunately, in these experiments, outcome measures were often restricted to grammaticality judgment tasks, limiting the generalizability of the findings to other linguistic functions. The study by Fiveash [65], which utilized alternative tasks and reported no significant effects, highlighted the need to employ multiple outcome measures.

It is worth noting that, unlike music-based and language-based rhythmic training, which engaged children in multiple sessions over periods ranging from two months to one year, rhythmic priming protocols typically involved a single training session. Previous studies have emphasized the role of training duration and intensity in producing significant outcomes [44, 52]. Interestingly, our review suggests that even short interventions can yield positive effects. Drawing on evidence from neuromodulation literature [76], we can hypothesize that repeated training sessions may produce more long-lasting effects, whereas single sessions are more likely to result in short-term after-effects. Follow-up data were obtained from a few studies using different time frames (3 months [60], 4 months [61], and 2 years [51]). Consistently, these studies showed that training benefits were maintained over time in reading speed [60] and reading accuracy [61] in children with DD, as well as in speech segmentation in TD children [51]. However, due to the limited number of studies and the heterogeneity of follow-up intervals, it is not possible to draw robust conclusions from these findings. The minimum effective training duration and the persistence of the effects over time should be systematically investigated in future studies.

When considering language-related or non-linguistic outcomes, music-based training appears to yield broader cognitive benefits. Evidence indicates that such training can positively impact several domains, including working memory [56, 58], visuospatial abilities [55], and inhibitory control [55]. In contrast, findings on language-related non-literacy skills after language-based rhythmic interventions are more inconsistent. Moreover, studies employing rhythmic priming sequences did not report significant improvements in non-linguistic domains. One possible explanation is that language-based and, even more, rhythmic priming training engage a narrower range of cognitive processes, as they do not involve the variety of activities included in music-based training. This variety likely facilitates greater generalizability. This suggests that music-based training may offer potential benefits that extend beyond linguistic functions.

Most music- and language-based training programs were conducted in group settings and included joint activities, whereas rhythmic priming sequences were administered individually. Although previous evidence has shown that group-based programs often achieve higher levels of attendance, adherence, and motivation than individually administered programs [77], indicating that social interaction can strengthen intervention effects, our review suggests that both types of settings yielded improvements. While it is plausible to hypothesize that the social context inherent to group activities may enhance children’s motivation and foster engagement, no studies have directly compared the effect of group-based versus individual rhythm interventions. Therefore, future studies should systematically compare these formats to clarify their differential impact on linguistic outcomes.

The training showed beneficial effects across the age range of 5 to 12 years. Post-training improvements were observed in both preschool- and school-aged children, in phonological processing (preschool: 50, 53; school: 51, 58–60) as well as syntactic processing (preschool: 66, 68; school: 28, 64, 65, 67, 69). This may suggest that the application of rhythmic training to boost linguistic functions could be effective across a broad range of developmental ages. This is in line with the few studies that have taken into account age as a covariate to explain the training effects and found that they were not influenced by age [63, 65]. From a methodological perspective, these studies underscored the importance of controlling for relevant covariates, such as chronological age and baseline reading abilities, to appropriately interpret the results. Moreover, only a limited number of studies considered the children’s past musical or rhythmic experience in participant recruitment, which may have influenced the sensitivity to the training effect [28, 51, 53, 56, 64, 68], although Patscheke et al. [53] did not reveal significant effects of either the children’s or their parents’ musical experience.

Importantly, while the efficacy of rhythmic training has been primarily tested in TD children, those studies that investigated its effects in children with language or reading disorders have also reported benefits. This finding reveals that rhythmic training is effective across both typical and atypical developmental populations and highlights its potential as a versatile intervention. As theorized by the Processing Rhythm in Speech and Music [32] and the Atypical Rhythm Risk Hypothesis [36] frameworks. The predictions of these models are further corroborated by improvements observed in language-related non-linguistic tasks involving entrainment skills, such as sensorimotor synchronization, temporal anisochrony discrimination, metrical perception, rhythm reproduction, and auditory attention [52, 55, 61, 62]. Moreover, improvements in phonological processing were often accompanied by gains in reading accuracy and fluency, confirming that strengthening phonological skills may directly support the development of reading abilities [43].

Some methodological aspects need to be improved before drawing any conclusive remarks. The majority of studies targeted either TD or clinical populations exclusively. Only a few studies, using a rhythmic priming approach, directly compared TD and clinical samples [28, 64–66]. Interestingly, in three out of these four studies, no significant interaction with the group was found, suggesting that the training effect was present in both populations. Of note, in one case [65] the training effect survived in the TD group only when results were controlled for age.

Another issue worth noting is that both music- and language-based training programs actively engage the child in exercises, whereas exposure to priming sequences typically involves passive participation. This difference in engagement may influence the observed effects, as previous studies have shown that passive control groups can overestimate training benefits [78]. While children can implicitly learn temporal regularities and easily translate this knowledge into motor and cognitive control adaptation [79, 80], active engagement may indeed further enhance learning outcomes. The impact of this difference in child involvement should be investigated in future studies, and active controls should be included to provide a more rigorous test of task-specific effects. This does not hold for the priming studies.

As discussed in the Introduction, the efficacy of rhythmic training in enhancing linguistic skills is rooted in its ability to facilitate synchronization between external inputs and internal neural oscillations [13, 15, 20]. Regular metric patterns, compared to irregular ones, help align internal oscillations with external cues, supporting the development of temporal predictions and leading to more efficient stimulus processing and response [81]. In other words, the mechanisms of statistical learning and neural entrainment may explain the superior efficacy of regular over irregular rhythms in enhancing linguistic performance. Indeed, regular rhythmic activities appear particularly effective in promoting temporal synchronization and anticipatory processing, thereby priming the cognitive system for linguistic input.

Several methodological caveats emerged from the review and may guide future investigations. First, the choice of control training is critical, as it significantly influences the interpretation of results. Age and pre-training outcome measures are fundamental variables that should be included in statistical analyses. Training-related changes should be inferred from a significant pre–post × group interaction rather than from within-group comparisons alone. Since the underlying mechanisms through which training may be effective could differ substantially between clinical and TD populations, their effects should also be statistically compared across typical and atypical groups. Employing a broader range of linguistic outcome measures would help identify which specific skills are most responsive to rhythmic training. In multi-session protocols, adherence to training and dropouts should be carefully monitored to minimize selection bias. Even though blinding is challenging in this kind of intervention studies, at least examiners should be blinded and independent from the trainer. Furthermore, the possibility of adapting the training intensity and requirements to each child’s individual skills deserves further attention (see [55]).

This review also presents some weaknesses that should be acknowledged. In particular, the high heterogeneity and methodological limitations of the included studies restrict the possibility of drawing definitive conclusions. Moreover, the review primarily relies on a descriptive analysis of study findings, as a quantitative synthesis would be unreliable due to the small number of comparable studies within each category.

From an applied standpoint, rhythmic training appears to be a flexible intervention tool. Most of the reviewed protocols rely on simple playful activities and require minimal equipment and resources. They have been implemented in both educational and clinical contexts and can be adapted to different age ranges and developmental profiles.

## Conclusions

The present review highlights a growing body of evidence supporting the efficacy of rhythmic training in enhancing language skills in children, including those with developmental language or reading difficulties. Specifically, studies employing music-based, language-based, or rhythmic priming training have demonstrated improvements in phonological processing, syntactic processing, and reading skills. Remarkably, these benefits were observed even in the absence of melodic or motor components, underscoring the unique contribution of rhythm and temporal regularity. The comparable effects of rhythmic training to those of validated phonological or reading interventions further support their efficacy.

While promising, the literature is limited by some methodological issues, such as a heterogeneous range of outcome measures and insufficient follow-up data. Future research should aim to identify the most effective rhythmic features, the linguistic functions most impacted, and the duration of observed effects.

Overall, rhythmic training emerges as a valuable approach for complementing programs aimed at supporting language and reading development.

## Supplementary Information

Supplementary Material 1.

## Data Availability

All data analysed during this study are included in this manuscript and its supplementary information file.

## Abbreviations

CASP: Critical Appraisal Skills Programme Randomised Controlled Trial Checklist
CG: Control group
DD: Developmental dyslexia
DLD: Developmental language disorder
EG: Experimental group
PA: Phonological Awareness
PRISMA: Preferred Reporting Items for Systematic Reviews and Meta-Analyses
RAN: Rapid Automatized Naming
RRT: Rhythmic Reading Training
TD: Typically developing
