# Supplementary material for: The efficacy of rhythmic training for enhancing language and reading skills in children: a systematic review
Source: BMC Psychol. 2026 Jul 11;14:1444. doi: 10.1186/s40359-026-04969-6 (PMC13640393; doi:10.1186/s40359-026-04969-6)
Supplement: Supplementary file 1 [file 40359_2026_4969_MOESM1_ESM.docx]

**Supplementary Material 1.**

**The efficacy of rhythmic training for enhancing language in children: a systematic review**

**Table S1** Study quality assessment checklist for studies with music-based, language-based rhythmic training (**a**), and rhythmic priming sequences (**b**). Y, yes (score 1); PF, partially fulfilled (score 0.5); N, no (score 0); NR, not reported (score 0); S, stratification; P, pseudo-randomization; C, by school class.

**a**

| **STUDY** | Bhide et al. (2013) [57] | Bonacina et al. (2015) [62] | Cancer et al. (2020) [59] | Cancer et al. (2022) [60] | Cancer et al. (2023) [61] | Degé & Schwarzer (2011) [50] | Flaugnacco et al. (2015) [58] | Francois et al. (2013) [51] | Frey et al. (2022) [52] | Harrison et al. (2018) [63] | Moreno et al. (2009) [56] | Patscheke et al. (2019) [53] | Rautenberg (2013) [54] | Zanto et al. (2024) [55] |
| --- | --- | --- | --- | --- | --- | --- | --- | --- | --- | --- | --- | --- | --- | --- |
| **Clear research question (cause-effect relationship)** | Y | Y | Y | Y | Y | Y | Y | Y | Y | Y | Y | Y | Y | Y |
| **Eligibility criteria for participants** | PF | Y | Y | Y | Y | NR | Y | PF | PF | Y | Y | Y | NR | NR |
| **Randomization of group assignment** | N | Y(S) | Y(P) | N | N | Y | Y(P) | Y(P) | Y(C) | Y | Y | Y | Y(C) | Y(C) |
| **Active control group** | Y | N | Y | N | Y | Y | Y | Y | Y | Y | Y | Y | Y | Y |
| **Groups matched for age or school grade** | Y | Y | Y | Y | Y | Y | Y | Y | Y | Y | Y | Y | Y | N |
| **Groups matched for non-verbal IQ** | Y | N | Y | Y | Y | Y | Y | Y | Y | N | Y | Y | Y | N |
| **Language and/or reading skills assessed at baseline** | Y | Y | Y | Y | Y | Y | Y | Y | Y | Y | Y | Y | Y | PF |
| **Sample size estimation** | N | N | Y | Y | N | N | Y | N | N | N | N | N | N | N |
| **Clearly defined training materials and procedures** | PF | Y | Y | Y | Y | Y | PF | PF | Y | Y | Y | Y | Y | Y |
| **Dropouts reported** | N | N | N | N | N | Y | Y | Y | N | N | Y | Y | N | Y |
| **Clearly defined outcome measures** | Y | Y | Y | Y | Y | Y | Y | Y | Y | Y | Y | Y | Y | Y |
| **Experimental vs. Control group differences at baseline statistically tested** | Y | Y | Y | N | Y | Y | Y | Y | Y | Y | Y | Y | Y | Y |
| **Pre vs. Post differences statistically tested** | Y | Y | Y | Y | Y | Y | Y | Y | Y | Y | Y | Y | Y | Y |
| **Pre-Post × Group interaction statistically tested** | Y | Y | Y | Y | Y | Y | Y | Y | Y | Y | Y | Y | NR | Y |
| **Score (%)** | 71.4 | 71.4 | 92.9 | 71.4 | 78.6 | 85.7 | 96.4 | 85.7 | 82.1 | 78.6 | 92.9 | 92.9 | 71.4 | 67.8 |

**b**

| **STUDY** | Bedoin et al. (2016) [64] | Canette et al. (2020) [67] | Chern et al. (2018)  [68] | Fiveash et al. (2020) [69] | Fiveash et al. (2023) [65] | Ladányi et al. (2021) [66] | Przybylski et al. (2013) [28] |
| --- | --- | --- | --- | --- | --- | --- | --- |
| **Clear research question (cause-effect relationship)** | Y | Y | Y | Y | Y | Y | Y |
| **Eligibility criteria for participants** | Y | N | Y | Y | Y | Y | Y |
| **Random priming sequences order** | Y | Y | Y | Y | Y | Y | Y |
| **Language and/or reading skills assessed at baseline** | Y | N | Y | N | Y | Y | Y |
| **Sample size estimation** | N | N | N | N | N | N | N |
| **Clearly defined training materials and procedures** | Y | Y | Y | Y | Y | Y | Y |
| **Clearly defined outcome measures** | Y | Y | Y | Y | Y | Y | Y |
| **Baseline condition** | Y | Y | N | N | N | Y | N |
| **Experimental vs. Control training differences statistically tested** | Y | Y | Y | Y | Y | Y | Y |
| **Score (%)** | 88.8 | 66.6 | 77.7 | 66.6 | 77.7 | 88.8 | 77.7 |
